# One-Dimensional Multi-Scale Domain Adaptive Network for Bearing-Fault Diagnosis under Varying Working Conditions

**DOI:** 10.3390/s20216039

**Published:** 2020-10-23

**Authors:** Kai Wang, Wei Zhao, Aidong Xu, Peng Zeng, Shunkun Yang

**Affiliations:** 1 State Key Laboratory of Robotics, Shenyang Institute of Automation, Chinese Academy of Sciences, Shenyang 110016, China; wangkai@sia.cn (K.W.); zhaowei@sia.cn (W.Z.); zp@sia.cn (P.Z.); 2 Key Laboratory of Networked Control System, Shenyang Institute of Automation, Chinese Academy of Sciences, Shenyang 110016, China; 3 Institutes for Robotics and Intelligent Manufacturing, Chinese Academy of Sciences, Shenyang 110169, China; 4 University of Chinese Academy of Sciences, Beijing 100049, China; 5 School of Reliability and Systems Engineering, Beihang University, Beijing 100191, China; ysk@buaa.edu.cn

**Keywords:** domain adaptation, fault diagnosis, convolutional neural network, multi-scale features, distribution discrepancy

## Abstract

Data-driven bearing-fault diagnosis methods have become a research hotspot recently. These methods have to meet two premises: (1) the distributions of the data to be tested and the training data are the same; (2) there are a large number of high-quality labeled data. However, machines usually work under different working conditions in practice, which challenges these prerequisites due to the fact that the data distributions under different working conditions are different. In this paper, the one-dimensional Multi-Scale Domain Adaptive Network (1D-MSDAN) is proposed to address this issue. The 1D-MSDAN is a kind of deep transfer model, which uses both feature adaptation and classifier adaptation to guide the multi-scale convolutional neural network to perform bearing-fault diagnosis under varying working conditions. Feature adaptation is performed by both multi-scale feature adaptation and multi-level feature adaptation, which helps in finding domain-invariant features by minimizing the distribution discrepancy between different working conditions by using the Multi-kernel Maximum Mean Discrepancy (MK-MMD). Furthermore, classifier adaptation is performed by entropy minimization in the target domain to bridge the source classifier and target classifier to further eliminate domain discrepancy. The Case Western Reserve University (CWRU) bearing database is used to validate the proposed 1D-MSDAN. The experimental results show that the diagnostic accuracy for the 12 transfer tasks performed by 1D-MSDAN was superior to that of the mainstream transfer learning models for bearing-fault diagnosis under variable working conditions. In addition, the transfer learning performance of 1D-MSDAN for multi-target domain adaptation and real industrial scenarios was also verified.

## 1. Introduction

Rotating machinery is one of the most important types of equipment in modern industrial applications. Bearings are frequently used mechanical parts in most rotating equipment and are the main source of faults of such equipment [1]. Georgoulas et al. [2] showed that bearing failures account for 44% of the total number of equipment failures. Thus, the reliability of bearings has become a critical issue and requires technical condition monitoring for fault diagnosis to improve the availability of equipment that incorporates bearings.

In recent years, with the accumulation of industrial big data technology and the rapid development of machine learning, especially deep learning, data-driven bearing-fault diagnosis methods have become a research hotspot. Deep learning architectures such as Convolutional Neural Networks (CNNs), Stacked Autoencoders (SAEs), Deep Belief Networks (DBNs), Recurrent Neural Networks (RNNs), and Generative Adversarial Networks (GANs) have been applied successfully in fault diagnosis [3]. Ince et al. [4] proposed a fast motor bearing-fault detection system using a one-dimensional convolutional neural network, which can fuse feature extraction and classification stages together. Wen et al. [5] proposed a new convolutional neural network method for fault diagnosis, which converts one-dimensional raw data into two-dimensional grayscale images for processing. To solve the impact of data imbalance on fault diagnosis, Wang et al. [6] and Mao et al. [7] use generative adversarial networks to generate the synthetic minority samples, and then, a stacked auto encoder is used for bearing-fault diagnosis. To reduce the computational complexity, Ma et al. [8] proposed a lightweight deep learning method for bearing-fault diagnosis based on a deep residual convolutional network. The algorithm can effectively improve the diagnostic accuracy, and the calculation speed is fast. Gan et al. [9] combined multiple DBNs to form a deep model for the feature learning of mechanical systems. The first layer identifies the location of the fault, and the second layer uses the results of the first layer to determine the size of the bearing fault. All the bearing-fault diagnosis methods mentioned above should meet the following two prerequisites: (1) there are a large number of high-quality labeled data, (2) the distributions of the data to be tested and the training data are the same. However, these prerequisites can seldom be met in practice. For example, machines usually have many different working conditions during operation, and the data distributions under different working conditions are different, which leads to the misclassification of traditional data-driven fault diagnosis methods. Furthermore, it is impractical to collect labeled training samples for each working condition and train the corresponding model, as required by traditional data-driven methods. Hao et al. [10] proposed an end-to-end solution for a one-dimensional convolutional long short-term memory network for bearing-fault diagnosis. In the proposed solution, spatial and temporal features are extracted and then combined to perform bearing-fault diagnosis more effectively. The method of Hao et al. has good adaptability to working conditions, but it cannot completely solve the impact of working condition differences. Domain adaptation (homogeneous transfer learning) [11] has been proposed to address this kind of problem recently. The principle of domain adaptation is eliminating the distribution discrepancy between the source domain (labeled data) and target domain (unlabeled data), so that the knowledge learned from the source domain can be effectively used in the target domain.

Currently, it is popular to use deep networks for domain adaptation because deep networks can automatically extract more expressive features. Compared with non-deep domain adaptation methods [12,13,14,15], deep domain adaptation methods [16,17,18,19] are easier to learn domain-invariant feature representations. Some deep domain adaptation methods have been developed to perform rolling bearing-fault diagnosis under variable working conditions. Li et al. [20] proposed a convolutional neural network-based domain adaptation for rolling bearing-fault diagnosis; the multi-layer and multi-kernel maximum mean discrepancies between the source and target domain data are minimized to address the domain discrepancy problem. Zhu et al. [21] proposed a deep domain adaptation method based on a two-dimensional convolutional neural network to address the problem, in which the vibration signals are converted into an image as an input sample and domain adaptation is performed at the last two layers. Zhang et al. [22,23] implemented domain adaptation by adding the adaptation of statistical features to the batch normalization layer. Zhang et al. [24] trained a domain adaptive convolutional neural network model to minimize the maximum mean squared error between the outputs of the two feature extractors so that features from the source and target domains had similar distributions after mapping. Zhang et al. [25] proposed a Wasserstein distance guided Multi-Adversarial network-based method, in which the learning process is to minimize the Wasserstein distance between the source domain and the target domain by using an adversarial training strategy. Qian et al. [26] built a fault diagnosis network that is robust with working condition variation based on high-order Kullback-Leibler (HKL) and transfer learning, wherein a sparse filter with HKL divergence is proposed for learning the domain-invariant features. In all the deep domain adaptation methods mentioned above, deep features are aligned for minimizing distribution discrepancy. However, these methods still face the following two challenges: (1) these methods extract domain-invariant features from a single feature extractor, which can only obtain partial information and leads to the result that the distribution discrepancy between different working conditions cannot be minimized to an acceptable range; (2) according to Long et al. [27], the domain-invariant features learned by feature adaptation can only reduce, instead of removing, the domain discrepancy. Namely, feature adaptation alone cannot fully meet the performance requirements for domain adaptation.

In order to address the challenges faced by the existing deep domain adaptation methods mentioned above, a one-dimensional Multi-Scale Domain Adaptive Network (1D-MSDAN) for the fault diagnosis of bearings under variable working conditions is proposed in this paper. The 1D-MSDAN model uses both feature adaptation and classifier adaptation to guide the multi-scale convolutional neural network. Feature adaptation is performed by both multi-scale feature adaptation and multi-level feature adaptation, which helps to find domain-invariant features by minimizing the distribution discrepancy between different working conditions by using the Multi-Kernel Maximum Mean Discrepancy (MK-MMD). At present, multi-scale feature adaptation methods have achieved good results in cross-domain image classification [28], while they have not been applied in fault diagnosis fields. Furthermore, classifier adaptation is performed in the proposed 1D-MSDAN by entropy minimization in the target domain to bridge the source classifier and target classifier to further eliminate domain discrepancy.

In general, the proposed 1D-MSDAN is the first to use both multi-scale feature adaptation and classifier adaptation for cross-domain fault diagnosis. The main contributions of this paper are summarized as follows:

(1) The 1D-MSDAN model is proposed for the fault diagnosis of motor bearings under different working conditions. Different domain-invariant features are learned from multi-scale convolutional neural networks, and the distribution discrepancy can thus be minimized by multi-scale and multi-level feature adaptation; in addition, the classifier adaptation bridges the source classifier and target classifier for further domain adaptation.
(2) The superiority of 1D-MSDAN is compared with some mainstream methods by implementing 12 transfer tasks on the Case Western Reserve University (CWRU) dataset, and feature visualization is used to further evaluate the superiority of the proposed 1D-MSDAN.
(3) A transfer model is established to simultaneously solve the fault diagnosis problem of two unlabeled conditions in order to further improve the transfer efficiency. The transfer efficiency is increased by 50% while ensuring accuracy.
(4) Different levels of Gaussian white noise are mixed with the data under testing to verify the effectiveness of 1D-MSDAN in real industrial scenarios.

The reminder of this paper is organized as follows. Section 2 describes the problem definition and some preliminary knowledge. In Section 3, the proposed 1D-MSDAN method is described in detail. The effectiveness and superiority of the proposed 1D-MSDAN are verified by several experiments in Section 4. Finally, the conclusions are presented in Section 5.

## 2. Preliminary Knowledge of Some Concepts

### 2.1. Problem Formalization

**Figure 1:**
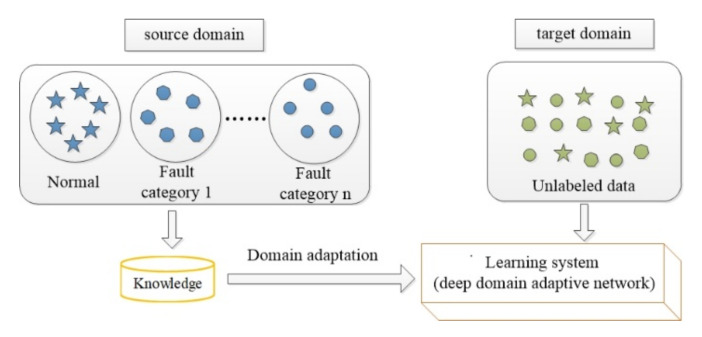
The learning process of domain adaptation.

In the field of fault diagnosis, the working conditions of machines are often changed. Different working conditions are defined as different domains. The working condition with labeled data is defined as the source domain $D_{s}={\left\{X_{i},y_{i}\right\}}_{i=1}^{n_{s}}$, and the working condition with unlabeled data is defined as the target domain $D_{t}={\left\{X_{j}\right\}}_{j=1}^{n_{t}}$. The numbers of source and target samples are $n_{s}$ and $n_{t}$, respectively. They have the same feature space, $\chi_{s}=\chi_{t}$, and categories, $y_{s}=y_{t}$, but have different distributions: $P_{s}(X_{s})\neq P_{t}(X_{t})$. Therefore, the fault diagnosis model of the source domain cannot be used directly to solve the fault diagnosis problem of the target domain. As shown in Figure 1, domain adaptation is to learn the label of the target domain with the help of the knowledge of the source domain, so as to solve the problem of fault diagnosis in the case of no label in the target domain.

### 2.2. Convolutional Neural Network

A convolutional neural network is a kind of neural network specially used to process data with a similar grid structure. For example, an image can be regarded as a two-dimensional pixel grid, and the one-dimensional vibration data can be regarded as a one-dimensional grid. In recent years, the CNN has been well applied in many fields, such as image classification [29], fault diagnosis [30], and so on. Convolutional neural networks use convolution operations instead of general matrix multiplication operations. Convolution operations help to improve machine learning systems through three important ideas: sparse interactions, parameter sharing, and equivariant representations [31].

#### 2.2.1. The Convolutional Layer

The function of the convolution layer is to extract the features from input data, and it contains multiple convolution kernels. Each element of a convolution kernel corresponds to a weight coefficient matrix and a bias vector, similar to a neuron of a feedforward neural network. The convolution operation is described as follows:

(1) $$y_{j}^{L}=f(\sum_{i}(y_{i}^{L-1}*K_{ij})+b_{j})$$

where f(•) is the activation function, $y_{j}^{L}$ is the local filtering result of the *j*-th filter, $K_{ij}$ is the *j*-th filter kernel, and $b_{j}$ is the bias.

#### 2.2.2. The Pooling Layer

The pooling layer is used to further adjust the output of the convolution layer. Convolution pooling has become a popular practice. The most common pooling operations are average pooling and max pooling. A combination of max pooling operation and average pooling operation is applied in this paper. The max pooling can reduce the deviation of the estimated mean caused by the convolution layer parameter error, while the average pooling is used to retain the overall characteristics. The general forms of max pooling and average pooling are described as follows:

(2) $$P_{j}^{L}=\max\left\{\left.y_{j}^{L*S:(L+1)*S}\right\}\right.$$

(3) $$P_{j}^{L}=\mathrm{mean}\left\{\left.y_{j}^{L*S:(L+1)*S}\right\}\right.$$

where *S* is the length of the sub-region and $P_{j}^{L}$ is the output of the *j*-th point.

#### 2.2.3. The Fully Connected Layer

The fully connected layer is the last part of the hidden layer of the convolutional neural network. The feature maps lose the spatial topology and are reshaped into a vector in the fully connected layer. Similar to a multi-layer perceptron, each neuron in a fully connected layer is fully connected to all the neurons in its previous layer. The form of a fully connected lay is described as follows:

(4) $$y_{}^{L}=f(\sum_{i}(W_{}^{L-1}*y_{}^{L-1})+b^{L})$$

where $W_{}^{L-1}$ and $b_{}^{L}$ are the weight matrix and bias vector, $y_{}^{L-1}$ is the input data from the upper layer, and f(•) is the activation function.

At last, the softmax logistic regression function is applied for the classification task after the last fully connected layer.

### 2.3. Maximum Mean Discrepancy

The Maximum Mean Discrepancy (*MMD*) measures the discrepancy between two distributions in the reproducing kernel Hilbert space (*RKHS*) [32], which is a kernel learning method. The *MMD* is widely used in transfer learning: the source domain data and the target domain data are embedded in a reproducing kernel Hilbert space (*RKHS*), and then, the mean distance between the two domains is calculated. The *MMD* between Ds and Dt is defined as

(5) $$MMD^{2}(\;D_{s},D_{t})={\left\|\frac{1}{n_{s}}\sum_{i=1}^{n_{s}}\phi (X_{i}^{s})-\frac{1}{n_{t}}\sum_{j=1}^{n_{t}}\phi (X_{j}^{t})\right\|}_{H}^{2}=\frac{1}{n_{s}{}^{2}}\sum_{i=1}^{n_{s}}\sum_{j=1}^{n_{s}}k(X_{i}^{s},X_{j}^{t})-\frac{2}{n_{s}n_{t}}\sum_{i=1}^{n_{s}}\sum_{j=1}^{n_{t}}k(X_{i}^{s},X_{j}^{t})+\frac{1}{n_{t}{}^{2}}\sum_{i=1}^{n_{t}}\sum_{j=1}^{n_{t}}k(X_{i}^{s},X_{j}^{t})$$

where *H* denotes the *RKHS*, and ϕ denotes the feature map with a Gaussian kernel function, $k(X_{i}^{s},X_{j}^{t})=<\phi (X_{i}^{s}),\phi (X_{j}^{t})>$.

The multi-kernel *MMD* (*MK-MMD*) [33] is an improvement of the *MMD* and can substantially enhance adaptation effectiveness compared to single kernel methods. Here, the domain discrepancy is further reduced using an optimal multi-kernel selection method for mean embedding matching [19]. At this point, $K(X_{i}^{s},X_{j}^{t})$ is redefined as the convex combination of multiple Gaussian kernels,

(6) $$K(X_{i}^{s},X_{j}^{t})=\sum_{u=1}^{m}\beta_{u}k_{u}(X_{i}^{s},X_{j}^{t}):\sum_{u=1}^{m}\beta_{u}=1,\beta_{u}\geq 0,\forall u$$

where β_u_ are the coefficients of every Gaussian kernel.

## 3. Proposed 1D-MSDAN for Bearing-Fault Diagnosis under Varying Working Conditions

**Figure 2:**
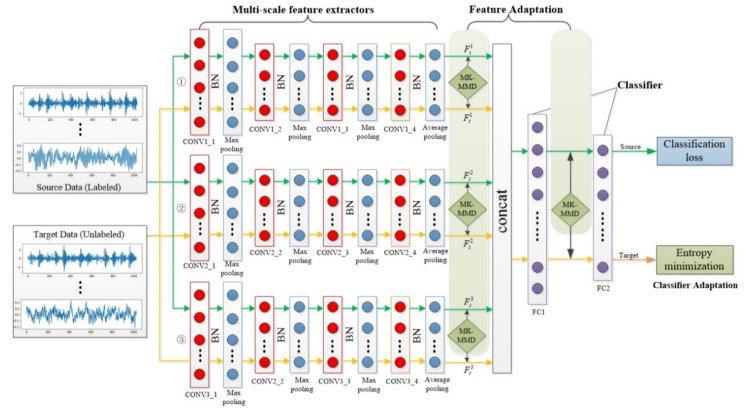
The architecture of the one-dimensional Multi-Scale Domain Adaptive Network (1D-MSDAN) model.

The architecture of the 1D-MSDAN model is shown in Figure 2. The proposed 1D-MSDAN consists of three parts: a multi-scale feature extractor, domain adaptation, and a classifier. The multi-scale feature extractor aims to learn the effective high-dimensional features of multiple scales to facilitate the classifier in judging bearing health conditions. Domain adaptation is implemented by multi-scale and multi-level feature adaptation and classifier adaptation to minimize the discrepancy in distribution between the source and target domains. The classifier is used for bearing health condition classification.

### 3.1. Multi-Scale Feature Learning

Multi-scale feature learning focuses on extracting multiple feature representations from samples through different structures. Multi-scale feature extractors and a classifier constitute a 1D multi-scale CNN. Different from the typical CNN architecture, the network has three feature extractors with different scales. These three feature extractors use different convolution kernels to extract features, which provides different sizes of receptive fields. The fusion of these multi-scale deep features provides opportunities for learning more compressive information for the purpose of domain adaptation.

The same original vibration signals, from source and target domains, are input to each feature extractor. As for each feature extractor, there are four convolutional layers in total for feature learning. The first convolutional layer uses the wide kernel proposed by Zhang Wei [22] for noise reduction, which can better suppress high-frequency noise. The other three convolution layers use convolution kernels with small sizes in order to learn deep feature representations. Meanwhile, the multi-layer small convolutional kernel makes the network deeper, which helps in obtaining the feature learning of the input signal and improving the model performance. In addition, because we need to learn different domain-invariant features between the two domains, the three feature extractors need to use different small convolution kernels. The receptive fields of different convolution kernels are different; that is to say, the information extracted by the three CNN channels will also be different. Besides, the combination of max pooling and average pooling is applied: max pooling can filter noise and reduce the interference of irrelevant information, while average pooling can prevent the loss of high-dimensional feature information. Furthermore, a batch normalization (BN) layer is also added behind each convolutional layer and before rectified linear units (ReLU) activation function. By whitening the input of each layer, the BN will take a step towards achieving a fixed input distribution, which will eliminate the adverse effects of internal covariate shifts [34]. To some extent, the BN accelerates the training of the network and solves the problem of overfitting and the dropout, and L2 regularization strategies are no longer used. At last, the fully connected layers, as the classifier, maps the learned deep feature representations to the label space through nonlinear mapping. The details of the 1D multi-scale CNN architecture are illustrated in Table 1.

### 3.2. Feature and Classification Adaptation

Due to the change in working conditions, the distributions of input data from different domains have discrepancies. The distribution discrepancy leads to a failure to correctly diagnose the health condition when the working condition of the machine changes. Both multi-scale and multi-level feature adaptation and classifier adaptation are used for solving the impact of distribution discrepancy.

#### 3.2.1. Multi-Scale and Multi-Level Feature Adaptation

Feature adaptation is used to reduce the impact of distribution discrepancies by learning domain invariant features. As for each feature extractor, the distribution discrepancy between the source and target domains is quantified by the *MK-MMD* between the output of the source and target domains. Domain-invariant features are thus learned by minimizing the *MK-MMD* of deep features between the source and target domains. Finally, multi-scale feature adaptation is implemented by learning domain-invariant features through multiple different feature extractors. Compared with traditional single-scale feature adaptation, multi-scale feature adaptation can learn more domain-invariant features from multiple feature extractors. In addition, multi-level feature adaptation is applied to learn more domain-invariant features. Namely, the *MK-MMD* is used in the first fully connected layer (FC1) to further calculate the distribution discrepancy. Thus, multi-scale and multi-level feature adaptation is defined as minimizing the following functions

(7) $$L_{feature}=0.5*\left(\sum_{i=1}^{3}MK-MMD^{2}(F_{s}^{i},F_{t}^{i})\right)+MK-MMD^{2}(FC_{s},FC_{t})$$

where $F_{s}^{i}$ and $F_{t}^{i}$ represent the output of the source domain and target domain data of the *i*-th feature extractor, respectively; $FC_{s}$ and $FC_{t}$ represent the output of FC1.

As the FC1 contains deep feature representations from three feature extractors, the weight coefficient of the *MK-MMD* between $F_{s}^{i}$ and $F_{t}^{i}$ is reduced to 0.5.

#### 3.2.2. Classifier Adaptation

Feature representations learned by deep networks can only reduce, instead of removing, the domain discrepancy [27]. Namely, although multi-scale and multi-level feature adaptations well reduce domain discrepancy, they cannot eliminate the mismatch in the classification model. Long et al. [27] assume that there is only a small perturbation function Δf(x) between the source classifier $f_{s}(x)$ and the target classifier $f_{t}(x)$; entropy minimization of the target data was used to optimize the parameters as the classifier adaptation to solve the perturbation function. In this paper, the entropy of the target data is defined as

(8) $$L_{entropy}=\frac{1}{n_{t}}\sum_{i=1}^{n_{t}}H(f_{t}(x_{i}^{t}))$$

where H(•) is the class-conditional distribution entropy function and $f_{t}(x)$ represents the output of FC2.

### 3.3. Optimization Objective and Training Strategy

The optimization objectives of the proposed 1D-MSDAN have three main parts: (1) minimizing the classification loss $L_{c}$ for the source domain data; (2) minimizing the feature discrepancy $L_{feature}$; (3) minimizing the entropy $L_{entropy}$ of the target data. Combining these three optimization objectives, the form of the eventual optimization object loss function is

(9) $$L=L_{c}+\lambda L_{feature}+\gamma L_{entropy}$$

where λ and γ are the tradeoff parameters; $L_{c}$ is the cross-entropy loss function as the classification loss.

(10) $$L_{c}=-\frac{1}{n_{s}}\sum_{i=1}^{n_{s}}\sum_{c=1}^{k}y^{\left(c\right)}*\log{\hat{y}}^{\left(c\right)}$$

where *k* is the number of categories, ${\hat{y}}^{\left(c\right)}$ is the probability under each label category, and $y^{\left(c\right)}$ is the real label.

Let $\theta_{1}$ be the parameters of the multi-scale feature extractors and the FC1 layer, and θ be all the network parameters. The eventual optimization cost function is further given as flows:

(11) $$L(\theta )=L_{c}(\theta )+\lambda L_{feature}(\theta_{1})+\gamma L_{entropy}(\theta )$$

After determining the optimization object, the back propagation algorithm and Adaptive moment estimation (Adam) [35] optimization algorithm are used to update the gradient and minimize the eventual optimization object (12). The training processes are described as flows.

(1) Initial θ with random values.
(2) Pre-training: update all the parameters by minimizing $L_{c}$ with the Adam optimization algorithm.
  (12) $$\theta \leftarrow \theta -\alpha \frac{\partial L_{c}(\theta )}{\theta }$$
(3) Repeat Step (2) until pre-training is finished.
(4) Domain adaptation training: update $\theta_{1}$ by minimizing $L_{feature}$, and update θ by minimizing $L_{c}$ and $L_{entropy}$.
  (13) $$\theta_{1}\leftarrow \theta_{1}-\alpha \frac{\partial L_{feature}(\theta_{1})}{\theta_{1}}$$
  (14) $$\theta \leftarrow \theta -\alpha \frac{\partial (L_{c}(\theta )+L_{entropy})}{\theta }$$
  where α is the learning rate.
(5) Repeat Step (4) until domain adaptation training is finished.

After the training, the domain discrepancy between the source and target domain is minimized. Thus, the 1D-MSDAN model can be used to classify the unlabeled samples in the target domain.

## 4. Case Study

As described in this section, in order to verify the effectiveness of the proposed 1D-MSDAN model, 12 transfer tasks were conducted on the Case Western Reserve University bearings dataset under different loads. Pytorch, a mainstream deep learning framework, was used to implement the 1D-MSDAN model, running on Ubuntu 16 (Canonical Ltd., London, UK) with a GTX1060 GPU (NVIDIA, Santa Clara, CA, USA).

### 4.1. Data Description and Parameter Setting

#### 4.1.1. Data Description

**Figure 3:**
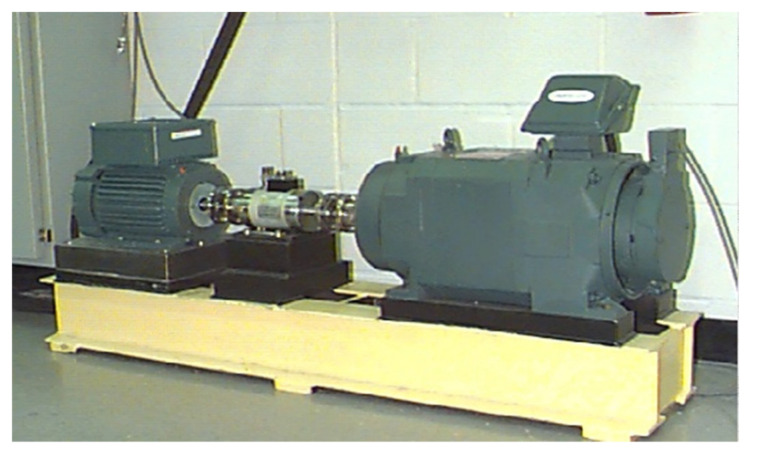
The experimental platform of Case Western Reserve University (CWRU).

The CWRU bearing dataset was collected from an experiment platform provided by the Case Western Reserve University; as shown in Figure 3, the experiment platform consists of a 2 hp motor (left), a torque transducer/encoder, a dynamometer, and control electronics [36]. There are a health type and three fault types, which are, namely, normal (NO), inner race fault (IF), outer race fault (OF), and roller fault (RF). There are three different levels of fault size (0.007 inches, 0.014 inches, and 0.021 inches) for each fault type when using electrical discharge machining (EDM). At a 12 K sampling frequency, the vibration signals are collected/obtained from the motor drive end under four load conditions (0 hp, 1 hp, 2 hp, and 3 hp). Data collected from four different loads are, respectively, defined as Domains A, B, C, and D. Thus, there are, in total, ten health categories with nine fault categories and one normal category for each domain. In our method, collecting 500 vibration signals from each category, the length of each vibration signal is 1024.

There are 5000 samples per domain, with ten categories. The detail is shown in Table 2. In order to more fully evaluate the robust performance of our method, twelve transfer tasks are set and each task is implemented five times: A→B, A→C, A→D, B→A, B→C, B→D, C→A, C→B, C→D, D→A, D→B, D→C. There are 80% of all the labeled data from the source domain, and unlabeled data in the target domain are in the training process. The remaining unlabeled data in the target domain are in the testing process.

#### 4.1.2. Parameter Setting

**Figure 4:**
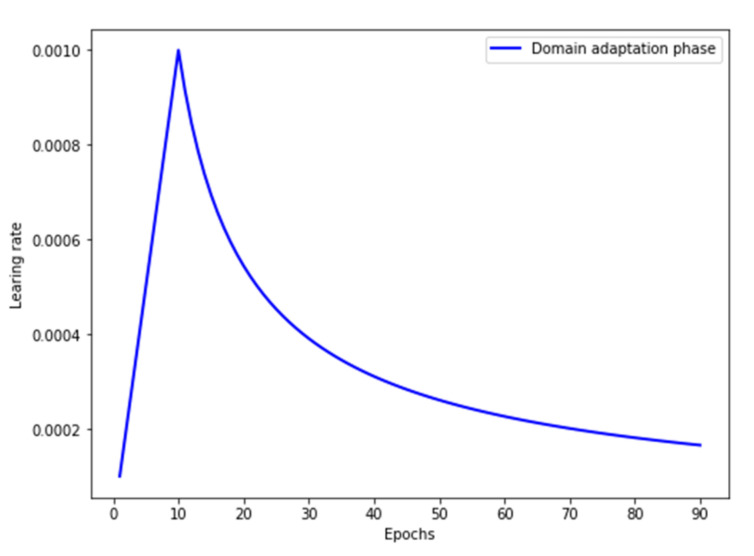
Learning rate of domain adaptation phase.

The 1D-MSDAN model is trained by using the Adam optimization algorithm, with a dynamic learning rate. The learning rate is set to a fixed value of 0.001, and the epochs are 10 in the pre-training phase. To maintain the stability of the model, the warmup strategy [37] is set for the learning rate and the epochs are 90 in the domain adaptation training phase. The warm-up strategy is to use a small learning rate to stabilize the model distribution and then use a large learning rate with dynamic decay to avoid falling into the local optimum. The learning rate setting of the domain adaptation phase is as shown in Figure 4; the learning rate increases linearly from 0.0001 to 0.001, and then, the learning rate decays dynamically [38]. The batch size is set as 50 due to the small dataset. The tradeoff parameter γ is set as 0.2. Due to different transfer tasks having different degrees of domain discrepancy, the tradeoff parameter λ between Domain A and Domain D is set to 1, and the remaining transfer tasks are set to 0.5.

### 4.2. Comparison with Other Transfer Learning Methods

To evaluate the superiority and efficacy of the proposed 1D-MSDAN, the transfer performance of the 1D-MSDAN is compared with some mainstream transfer learning algorithms in the field of bearing-fault diagnosis.

- **Ensemble TICNN**: Zhang et al. [23] proposed TICNN, inspired by AdaBN [39]. TICNN replaces the batch normalization (BN) statistics from the source data with those from the target data to reduce the distribution discrepancy. In addition, Ensemble TICNN adds ensemble learning to improve the stability of the algorithm.
- **SF-SOF-HKL**: Inspired by moment discrepancy and Kullback-Leibler (KL) divergence, Qian et al. [26] proposed using high-order KL (HKL) divergence to align the high-order moments of the domain-specific distributions. Sparse filtering with HKL divergence (SF-HKL) can learn both discriminative and shared features between the source and target domains. Besides, Qian et al. validated that softmax regression with HKL divergence (SOF-HKL) can further improve performance.
- **DACNN**: Zhang et al. [24] proposed DACNN, which consists of three parts: a source feature extractor, a target feature extractor, and a label classifier. Like our approach, DACNN uses a two-stage training process: First, it uses pre-training to obtain the fault discriminative features. Then, the target feature extractor is trained to minimize the squared MMD. Different from in other domain adaptation models, the layers between the source and target feature extractor are partially untied in the training.
- **WDMAN**: Zhang et al. [25] proposed an adversarial training strategy for Multi-Adversarial networks guided by the Wasserstein distance to learn the domain-invariant features between the source and target domains. This method is inspired by the Generative Adversarial Network (GAN) [40], and its purpose is to learn a generator to generate fake images that are indistinguishable from real images.

The comparison results for fault diagnostic accuracy are shown in Table 3. It can be observed that the average accuracy of the proposed 1D-MSDAN reaches 99.97% and is higher than that of other methods. Multi-scale and multi-level feature adaptation enables our model to learn more domain-invariant features than other methods. From the perspective of twelve tasks, the robustness and superiority of the proposed 1D-MSDAN is optimal, followed by WDMAN. As for SF-SOF-HKL, although it performs very well on some specific tasks, the average accuracy for all tasks is poor, which shows that the robustness and generalization of SF-SOF-HKL are not outstanding. In addition, although DACNN does not implement all the tasks, the average accuracy of the implemented tasks is close to that for WDMAN. Finally, the result for Ensemble TICNN is relatively poor, indicating that it is difficult to learn domain-invariant features only by changing the BN layers.

**Figure 5:**
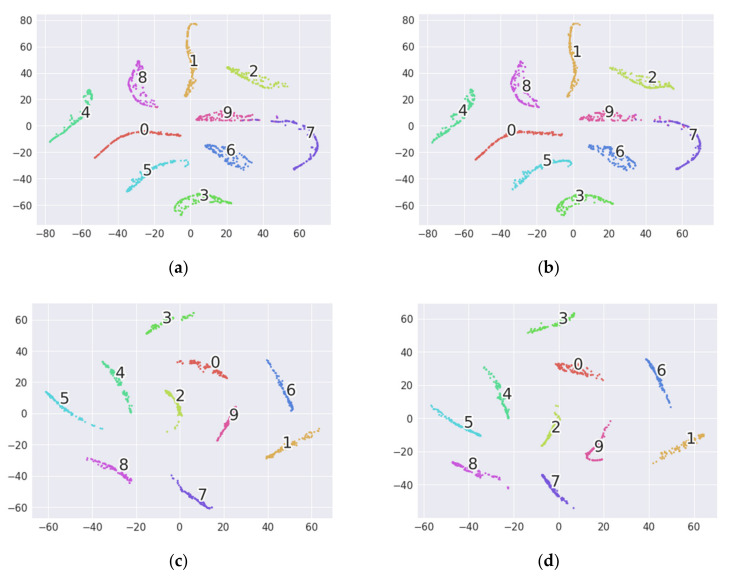
The two-dimensional visualization of the second full-connection layer of transfer task D→B. (**a**) WDMAN: source. (**b**) WDMAN: target. (**c**) 1D-MSDAN: source. (**d**) 1D-MSDAN: target. Category 0: No, Category 1: 0.007/IF, Category 2: 0.014/IF, Category 3: 0.021/IF, Category 4: 0.007/OF, Category 5: 0.014/OF, Category 6: 0.021/OF, Category 7: 0.007/RF, Category 8: 0.014/RF, Category 9: 0.021/RF.

Feature visualization is used to further evaluate the superiority of the proposed 1D-MSDAN. The t-distributed stochastic neighbor embedding (t-SNE) [41] technology is introduced to non-linearly reduce the output of the second fully connected layer to two dimensions for visualization. Since the performance of WDMAN is close to that of our proposed 1D-MSDAN, the feature visualization of WDMAN and 1D-MSDAN is implemented. Taking Task D→B as an example, the visualization results for WDMAN and 1D-MSDAN are shown in Figure 5. As can be observed from Figure 5, on the whole, the source and target domain data are aligned by both of the methods. However, Category 7 and Category 9 of WDMAN are not well clustered, which will cause some of the data of WDMAN’s Category 7 and Category 9 to be misclassified. This is consistent with the data in Table 3, which show that the accuracy of WDMAN is only 98.27% for D–B. On the contrary, it can be observed from Figure 5c,d that our method can achieve an effective result for domain adaptation, namely, each category can be clustered well.

**Figure 6:**
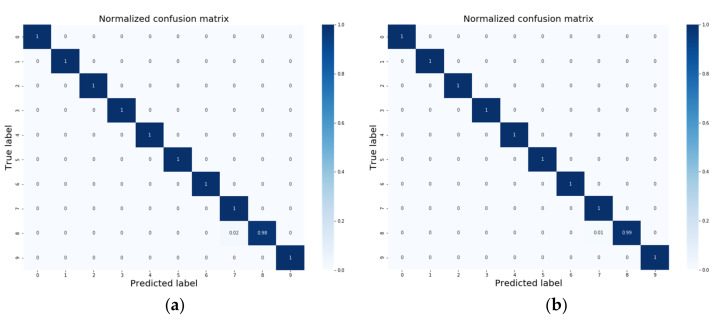
Confusion matrices of Tasks D→A: (**a**) Accuracy: 99.80%; (**b**) Accuracy: 99.90%. Category 0: No, Category 1: 0.007/IF, Category 2: 0.014/IF, Category 3: 0.021/IF, Category 4: 0.007/OF, Category 5: 0.014/OF, Category 6: 0.021/OF, Category 7: 0.007/RF, Category 8: 0.014/RF, Category 9: 0.021/RF.

To further show the results, the confusion matrix of Task D→A is shown in Figure 6; the reason for selecting Task D→A is that its diagnostic accuracy is the lowest among the others in Table 3. In the five experiments performed on Task D→A, the results were 99.80% (two samples are misclassified) for three times and 99.90% (one sample is misclassified) for two times. As shown in Figure 6, the samples of Category 8 were misclassified into Category 7, which is mainly due to the same fault type and the large discrepancy between working conditions D and A.

### 4.3. Verification of Multi-Target Domain Adaptation

**Figure 7:**
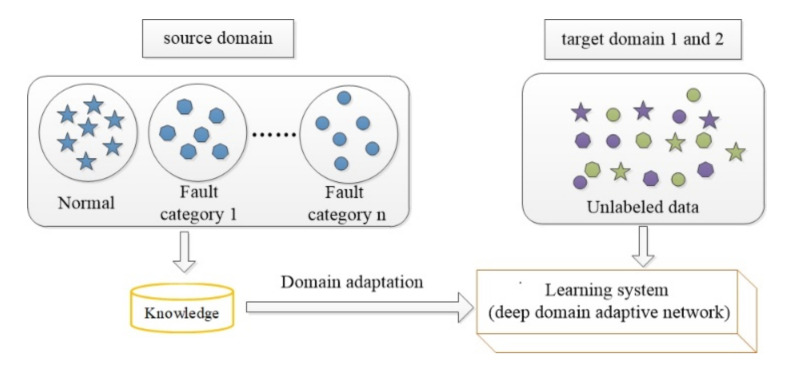
The learning process of multi-target domain adaptation.

In a traditional domain adaptation network, the transfer task is usually one-to-one, namely, each transfer task has one unique target domain. To improve the transfer efficiency and computing resource utilization, the transfer task setting is changed from one-to-one to one-to-two. As shown in Figure 7, domain adaptation is to learn the label of two target domains with the help of the knowledge of the source domain. At this point, the target domain data become unlabeled data of the two working conditions, and a transfer task solves the unlabeled fault diagnosis of the two working conditions. Six new transfer tasks are set: A→B + C, B→A + C, B→C + D, C→B + A, C→B + D, and D→B + C.

To avoid adding unnecessary computing resources, the total number of data in the target domain is the same as in the original task. The data of each working condition in the target domain become half of those in the original task. The number of training parameters and training time of each task are the same as in the original task. This is equivalent to the original two tasks becoming a task, resulting in greatly improved efficiency.

**Figure 8:**
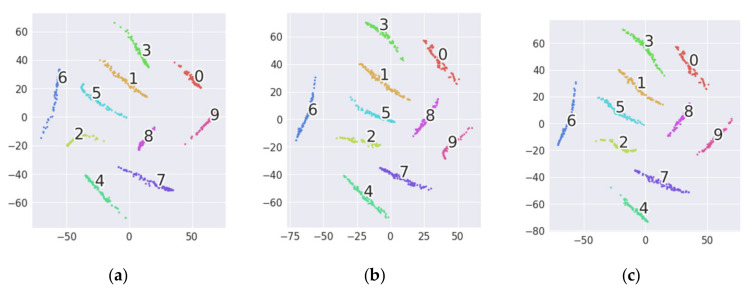
The two-dimensional visualization of the second full-connection layer of transfer task A→B + C. (**a**) 1D-MSDAN: source. (**b**) 1D-MSDAN: Target 1. (**c**) 1D-MSDAN: Target 2. Category 0: No, Category 1: 0.007/IF, Category 2: 0.014/IF, Category 3: 0.021/IF, Category 4: 0.007/OF, Category 5: 0.014/OF, Category 6: 0.021/OF, Category 7: 0.007/RF, Category 8: 0.014/RF, Category 9: 0.021/RF.

The results of the new tasks are shown in Table 4. From the results, it can be found that the accuracy of the new task is almost the same as that of the original task. Similarly, feature visualization is used to further evaluate the effect of the multi-target domain adaptation for fault diagnosis. Take Task A→B + C as an example, and the visualization results of 1D-MSDAN are shown in Figure 7. It can be observed from Figure 8a–c that the data in the three domains are well aligned, and each class is clustered correctly after multi-domain adaptation. According to the visualization results, all three domains have learned domain-invariant features, which indicates that multi-target domain adaptation is effective.

### 4.4. Verification of Real Industrial Scenarios

**Figure 9:**
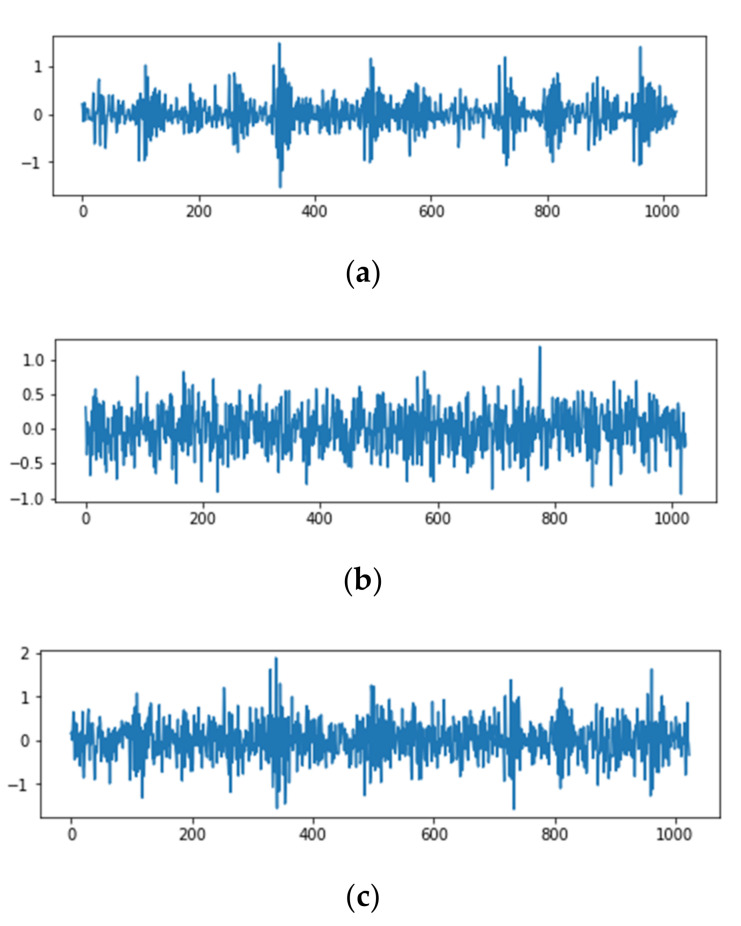
The original vibration signal is mixed with 1 dB of noise. (**a**) The original vibration signal. (**b**) Gaussian white noise of 1 dB. (**c**) The mixed signal.

In real industrial scenarios, machines usually work in a noisy working environment and the collected signals often have noise, which brings difficulties to fault diagnosis for machines. It is more meaningful to correctly judge machine health conditions with noise interference. In order to simulate different real industrial scenarios, we add white Gaussian noise with different signal-to-noise ratios (SNRs) to the original samples to test our model. The SNR is the ratio of useful signal power to noise power. The SNR is defined as follows:

(15) $$SNR(dB)=10\log_{10}(\frac{P_{signal}}{P_{noise}})=20\log_{10}(\frac{A_{signal}}{A_{noise}})$$

where $P_{signal}$ and $P_{noise}$ are the power of the signal and noise, respectively; $A_{signal}$ and $A_{noise}$ are the amplitudes of the signal and noise, respectively. As shown in Figure 9, there is a mixed signal with Gaussian white noise of 1 dB.

As described in this section, white Gaussian noise of 0 dB, 1 dB, and 2 dB is added to the data of the CWRU bearing dataset. Twelve transfer tasks are implemented again under each level of noise. The fault diagnosis results under each level of noise for 1D-MSDAN are shown in Table 5. From the results of Table 5, it can be seen that the diagnosis results for 1D-MSDAN still maintain a high accuracy after mixing different levels of noise. Therefore, these results demonstrate 1D-MSDAN still has good generalization performance in real industrial scenarios.

## 5. Conclusions

Bearing-fault diagnosis plays important roles in improving the availability of rotating machinery. Developing bearing-fault diagnosis models under varying working conditions is the key to applying fault diagnosis techniques in practice. In this paper, a deep transfer model, 1D-MSDAN, is proposed to achieve rolling bearing-fault diagnosis under variable working conditions. The core of the proposed model is (1) multiple feature extractors are used to learn multiple deep features of different scales, and thereby, multi-scale and multi-level feature adaptation are used to minimize domain discrepancy, and (2) considering that feature adaptation cannot completely eliminate domain discrepancy, the entropy minimization of the target domain is used as a classifier adaptation to further eliminate domain discrepancy based on feature adaptation.

Twelve transfer tasks on the CWRU dataset were performed with 1D-MSDAN and mainstream transfer learning algorithms, such as WDMAN, DACNN, SF-SOF-HKL, and Ensemble TICNN, and the results showed that the diagnostic accuracy of 1D-MSDAN is superior to that of the other methods. In addition, multi-target domain adaptation tasks in which the target domain data were from two operating conditions were performed with 1D-MSDAN, and the results showed that the diagnostic accuracy was almost the same as in the single-target domain adaptation tasks. Finally, 1D-MSDAN was verified under real industrial scenarios in which the data of the CWRU dataset were mixed with different levels of noise to simulate different real industrial situations, and the results showed that the diagnostic accuracy of 1D-MSDAN was still high.

The 1D-MSDAN still has room for improvement. At present, only the *MK-MMD* is used as a strategy to measure the distribution discrepancy. Since the *MMD* is highly sensitive to kernel selection, the selected kernels are not guaranteed to be suitable for all tasks. In addition, *MMD* computing needs to embed data into a reproducing kernel Hilbert space, which is a relatively large amount of computing. In future research, multiple measurement strategies will be tried to quantify the distribution discrepancy. Furthermore, measurement strategies with high calculation efficiency need to be developed.

## Figures and Tables

**Table 1:** Details of 1D multi-scale Convolutional Neural Network (CNN).

Module	Layer	Parameters	Activation Function	Output Size
Input	Input	/	/	1024 × 1
Feature extractor 1	Convolution 1_1	Kernel_size = 20 × 1, stride = 2	ReLU	512 × 16
	Max pooling 1_1	Kernel_size = 2 × 1, stride = 2	/	256 × 16
	Convolution 1_2	Kernel_size = 5 × 1, stride = 1	ReLU	256 × 32
	Max pooling 1_2	Kernel_size = 2 × 1, stride = 2	/	128 × 32
	Convolution 1_3	Kernel_size = 5 × 1, stride = 1	ReLU	128 × 64
	Max pooling 1_3	Kernel_size = 2 × 1, stride = 2	/	64 × 64
	Convolution 1_4	Kernel_size = 5 × 1, stride = 1	ReLU	64 × 64
	Average pooling 1_4	Kernel_size = 2 × 1, stride = 2	/	32 × 64
Feature extractor 2	Convolution 2_1	Kernel_size = 20 × 1, stride = 2	ReLU	512 × 16
	Max pooling 2_1	Kernel_size = 2 × 1, stride = 2	/	256 × 16
	Convolution 2_2	Kernel_size = 3 × 1, stride = 1	ReLU	256 × 32
	Max pooling 2_2	Kernel_size = 2 × 1, stride = 2	/	128 × 32
	Convolution 2_3	Kernel_size = 3 × 1, stride = 1	ReLU	128 × 64
	Max pooling 2_3	Kernel_size = 2 × 1, stride = 2	/	64 × 64
	Convolution 2_4	Kernel_size = 3 × 1, stride = 1	ReLU	64 × 64
	Average pooling 2_4	Kernel_size = 2 × 1, stride = 2	/	32 × 64
Feature extractor 3	Convolution 3_1	Kernel_size = 20 × 1, stride = 2	ReLU	512 × 16
	Max pooling 3_1	Kernel_size = 2 × 1, stride = 2	/	256 × 16
	Convolution 3_2	Kernel_size = 1 × 1, stride = 1	ReLU	256 × 32
	Max pooling 3_2	Kernel_size = 2 × 1, stride = 2	/	128 × 32
	Convolution 3_3	Kernel_size = 1 × 1, stride = 1	ReLU	128 × 64
	Max pooling 3_3	Kernel_size = 2 × 1, stride = 2	/	64 × 64
	Convolution 3_4	Kernel_size = 1 × 1, stride = 1	ReLU	64 × 64
	Average pooling 3_4	Kernel_size = 2 × 1, stride = 2	/	32 × 64
Classifier	Fully connected 1	Weights = 64 × 96, bias = 1024	ReLU	1014 × 1
	Fully connected 2	Weights = 1024 × 10, bias = 10	Softmax	10 × 1

**Table 2:** Description of dataset.

Domain	Operation Conditions	Number of Samples	Number of Categories
A	0 HP	5000	10
B	1 HP	5000	10
C	2 HP	5000	10
D	3 HP	5000	10

**Table 3:** Diagnostic accuracy (%) for 12 tasks.

	Ensemble TICNN [23]	SF-SOF-HKL [26]	DACNN [24]	WDMAN [25]	1D-MSDAN
A→B	_	99.80%	_	99.73%	**100.00%**
A→C	_	87.56%	_	99.67%	**100.00%**
A→D	_	99.70%	_	**100.00%**	**100.00%**
B→A	_	99.86%	_	99.13%	**99.95%**
B→C	99.50%	99.59%	**100.00%**	**100.00%**	**100.00%**
B→D	91.10%	95.50%	99.69%	99.93%	**100.00%**
C→A	_	88.50%	_	98.53%	**99.90%**
C→B	97.60%	99.23%	**100.00%**	99.80%	**100.00%**
C→D	99.40%	98.16%	99.90%	**100.00%**	**100.00%**
D→A	_	**100.00%**	_	98.07%	99.86%
D→B	90.20%	95.17%	97.98%	98.27%	**99.90%**
D→C	98.7%	97.81%	**100.00%**	99.53%	**100.00%**
AVG	_	96.74%	_	99.39%	**99.97%**

**Table 4:** The diagnosis accuracy (%) of multi-target domain adaptation.

Task	Parameter	Target 1	Target 2
A→B + C	*λ* = 1, *γ* = 0.2	100.00%	100.00%
B→A + C	*λ* = 1, *γ* = 0.2	99.90%	100.00%
B→C + D	*λ* = 1, *γ* = 0.2	100.00%	100.00%
C→B + A	*λ* = 1, *γ* = 0.2	99.80%	99.80%
C→B + D	*λ* = 1, *γ* = 0.2	100.00%	100.00%
D→B + C	*λ* = 1, *γ* = 0.2	99.30%	100.00%

**Table 5:** The diagnosis accuracy (%) of 1D-MSDAN under different noisy environments.

Task	SNR = 1	SNR = 2	SNR = 3	No Noise
A→B	98.76%	99.70%	99.70%	100.00%
A→C	99.60%	99.85%	99.90%	100.00%
A→D	99.70%	99.68%	99.92%	100.00%
B→A	98.50%	99.30%	99.10%	99.70%
B→C	99.68%	99.95%	99.95%	100.00%
B→D	99.01%	99.70%	99.85%	100.00%
C→A	99.08%	99.34%	99.40%	99.90%
C→B	97.44%	97.20%	97.20%	100.00%
C→D	100.00%	99.80%	100.00%	100.00%
D→A	99.14%	99.60%	99.70%	99.70%
D→B	97.24%	97.10%	97.10%	99.90%
D→C	100.00%	100.00%	99.85%	100.00%
AVG	99.01%	99.27%	99.31%	99.97%
